# Determinants of accounting information system effectiveness and moderating role of external consultants: Empirical research in the Ben Tre Province of Vietnam

**DOI:** 10.1016/j.heliyon.2024.e28847

**Published:** 2024-03-29

**Authors:** Huu Thien Nguyen, Ramayah T, Qian Long Kweh, Phuong Thi Kim Tran, Hieu Tran Duong Minh

**Affiliations:** a Faculty of Accounting, Ton Duc Thang University, Ho Chi Minh City, Viet Nam; b School of Management, Universiti Sains Malaysia, Minden, 11800, Penang, Malaysia; c Department of Information Technology & Management, Daffodil International University, Birulia, Bangladesh; d Department of Management, Sunway University Business School, 47500, Petaling Jaya, Selangor, Malaysia; e University Center for Research & Development (UCRD), Chandigarh University, Ludhiana, 140413, Punjab, India; f Faculty of Economics and Business, Universitas Indonesia (UI), Depok City, West Java, 16424, Indonesia; g The University of Jordan (UJ), Aljubeiha, Amman, Jordan; h Azman Hashim International Business School, Universiti Teknologi Malaysia (UTM), Kuala Lumpur, Malaysia; i School of Management, Canadian University Dubai, United Arab Emirates; j Faculty of Accounting – Finance, Dong Nai Technology University, Bien Hoa City, Viet Nam; k Merck Vietnam Co., Ltd, 106 Nguyen Van Troi, Phu Nhuan District, Ho Chi Minh City, Viet Nam

**Keywords:** AIS effectiveness, Determinant of AIS, External consultant, Information quality, System complexity

## Abstract

The characteristics of accounting information systems (AISs) within organizations and the factors affecting their effectiveness are investigated in this study. In particular, how external consultants moderate the relationship between the determinants and AIS effectiveness is examined. A total of 167 agricultural companies in the Ben Tre Province of Vietnam were surveyed using a regression-based partial least squares structural equation model. Then, the influence of these determinants on AIS effectiveness was evaluated. The findings showed that managers' involvement and managers’ accounting knowledge positively affect AIS effectiveness. Furthermore, the involvement and knowledge of managers are mitigated by external consultants, which reduces the negative influence of such involvement on AIS effectiveness. This study aims to contribute to the body of knowledge on the determinants affecting AIS effectiveness by providing agricultural companies in Ben Tre and Vietnam with insights into the effectiveness of their respective AIS activities.

## 1 Introduction

At present, businesses use accounting information systems (AISs) to maintain their competitiveness and boost their organizational performance [[1], [2], [3]]. Effective AISs can improve company efficiency, decision making, and cost savings [[4], [5], [6]]. The effectiveness of AISs depends on a variety of factors, including system complexity, participation of managers in AIS operations, and knowledge of managers of AIS and accounting [4,7]. However, external consultants can also influence the implementation and effectiveness of AIS [2,8]. Thus, research on the determinants of AIS effectiveness and the moderating role of external consultants presents an opportunity to expand the perception of the factors that influence the effectiveness of AIS [3,9,10].

System complexity affects the effectiveness of AIS, especially the accuracy and reliability of the data it produces [9,11,12]; however, complex systems may need additional resources for effective maintenance and operation. Errors or breakdowns may occur when these issues are not adequately managed [4,13]. Complexity also affects the training and expertise requirements of an effective AIS. Training may be costly and time consuming and require iterations to keep pace with system changes or external factors [3,14]. Firms need flexible and adaptive AISs to overcome these changes and continue producing accurate and reliable financial information [5].

The effectiveness of AIS is affected by managers' involvement in system operations. In this study, a “manager” specifically refers to individuals with financial and accounting functions in AIS operations. They typically hold the positions of director, vice director, chief accountant, or general accountant. These measures ensure that the AIS is fully integrated into a firm's operations and can produce reliable and accurate data [15,16]. The involvement of these individuals also helps to ensure that the AIS can be used for its fullest potential [[17], [18], [19], [20]]. Managers help to identify new ways of using the system or necessary improvements and identify training or resource requirements to maximize the system's benefits [20]. More importantly, managers help foster a culture of accountability and transparency within the firm [16,21]. Accountability and transparency build trust among stakeholders and improve the overall reputation of the firm.

Managers' knowledge of accounting and AIS features affect the effective operation of AIS. With sufficient knowledge, managers can efficiently ensure accurate accounting information and navigate the financial implications of business transactions [4,22]. As accounting knowledge continues to improve, managers become more efficient at sharing feedback with AIS developers, thus improving the system's functionality and meeting the firm's specific needs [23,24]. Furthermore, managers who are knowledgeable about AIS can use the system to identify potential areas of fraud, waste, or inefficiency and implement mitigation measures against these risks [25,26]. The knowledge of AIS combined with accounting enables managers to interpret the produced financial data, ensure their accuracy and reliability, and identify potential risks and novel opportunities for the organization [3,22,27].

External consultants considerably affect the implementation and effectiveness of AIS [4,17]. They also offer training and support to managers and employees who need to gain knowledge and skills in AIS operation [28,29]. Third-party evaluators are expected to act objectively when assessing the efficiency and effectiveness of the AIS of firms, especially when identifying system gaps and recommending points of improvement (A [4,30]). Owing to the expertise, objectivity, and a fresh perspective on AIS implementation of external consultants, they become beneficial to firms [3,4].

Firms benefit extensively from external consulting services in terms of AIS effectiveness and performance. However, research on the moderating role of external consultants is still needed [31,32]. External consultants play an important moderating role in enhancing AIS effectiveness by facilitating knowledge transfer, providing valuable insights, and promoting organizational learning (A [5,86]). Furthermore, an effective AIS requires substantial resources and expertise for implementation and maintenance [17,33,34]. External consultants provide fresh perspectives and new ideas, indicating novel approaches to innovation and performance evaluation [4,35]. However, the success of external consulting services cannot be easily guaranteed. Managers must carefully select external consultants to fully realize their potential advantages for firms [28,29,35]. The selection process includes assessing the consultant's expertise, whether the consultant is fit with the organization's culture, and problem-solving capabilities.

Companies in the Ben Tre Province of Vietnam were selected as the samples of empirical research samples. This study focused on the determinants of AIS effectiveness and the moderating role of external consultants. Several moderating factors are considered. First, Ben Tre Province is located in the Mekong Delta region of southern Vietnam. The province has a plain terrain with many rivers and canals flowing through and a rich and diverse natural environment for developing agriculture and fisheries. Therefore, most enterprises operate in the fields of agriculture, agricultural product processing, and food processing. Second, the transportation system includes roads and waterways, mainly waterways with low transportation costs, helping businesses have cost advantages in goods supply activities. Third, Ben Tre Province is implementing a national digital transformation program for 2022–2025. And according to Decision No. 1998/QD-UBND of the People's Committee of Ben Tre Province, all organizations across the province must implement digital transformation. The project is to support and improve information technology capacity for businesses in the area to share and promptly provide data. Thus, these characteristics could provide a unique context for studying AIS effectiveness.

Although considerable research has been conducted on AIS effectiveness, the current literature lacks sufficient exploration of the influence of specific determinants, including factors, such as system complexity, managerial involvement, and knowledge of AIS and accounting, on the effectiveness of AIS [3,5]. Thus, further research is needed to consider the relationship between these determinants and AIS effectiveness [4,5,7]. Furthermore, the potential moderating role of external experts must be adequately examined. Thus, in this study, we aim to (1) examine the relationship between AIS effectiveness and its determinants, including the complexity of AIS, the involvement of managers in its operations, and their knowledge of AIS and accounting, and (2) investigate the moderating role of external consultants on this relationship.

This study considerably contributes to academics and practitioners by exploring these relationships. First, it is helpful for organizations in streamlining processes, improving decision-making, reducing errors, and providing timely and accurate financial information. Second, it contributes to the theoretical understanding of AIS effectiveness by exploring and identifying the determinants affecting AIS effectiveness and the nature and extent of their influence. This result leads to the development of theoretical frameworks for future research and practical recommendations. Third, practitioners can apply the findings to improve the design and implementation of AIS in their organizations. This adoption improves organizational performance, reduces costs, and enhances competitiveness. Furthermore, the results of the study help organizations keep pace with the changing technological landscape and adapt their accounting systems accordingly.

The remainder of this paper is structured as follows: The first section provides a literature review and outlines the fundamental principles of the investigation. The second section presents the theoretical model and the links between its elements, the study technique, and the data collection methods. Next, the results are discussed in the third section, and their implications are explored. The final section contains the study's conclusions, limitations, and suggestions for future research.

## 2 Literature review

Successful information system models [36] are investigated to explore the factors influencing the effectiveness of AIS. In these models, the effectiveness of AIS is measured by its influence on staff and organizational performance. AIS effectiveness is reportedly affected by the three factors of system quality, information quality, and user satisfaction, which are considered determinants of AIS effectiveness [36]. Then, the model was used to investigate the dimensions of the influencing factors of AIS effectiveness. Overall, this model can help provide a robust theoretical framework for understanding the distinct relationships of AIS effectiveness among agricultural companies in Ben Tre Province, Vietnam. The determinants of AIS effectiveness can be described as follows:

***System Quality***: AIS complexity is a determinant of system quality. Complexity is defined as the degree to which a system can support functional and user task requirements [36]. As complex systems are challenging to manage, they may also negatively affect the satisfaction of users [[37], [38], [39]].

***Information Quality***: Managers’ involvement in AIS operation and their knowledge of AIS and accounting are determinants of information quality. Information quality is defined as the degree to which a system can guarantee accurate, timely, and relevant information to users [36]. User involvement and knowledge enhance the quality of information provided by the AIS [4,6,40].

***User Satisfaction***: External consultants help mediate the relationship between AIS effectiveness and user satisfaction. User satisfaction is the degree to which users perceive a system to meet their needs and expectations [36]. The involvement of external consultants can enhance user satisfaction by providing expertise and support for AIS implementation and maintenance [4,38,41,42].

On the basis of the theoretical framework, system complexity affects the effectiveness of AIS, the involvement of managers in AIS operations, and their knowledge of AIS and accounting. The framework further indicates that the relationship between AIS effectiveness and its determinants is strengthened by the involvement of external consultants.

### 2.1 AIS complexity

An AIS is critical to organizational functions because it provides decision-making information to managers. However, the complexity of such systems hinders the practical use of AIS; it also weakens user satisfaction and organizational performance [38,41]. Understanding the influence of system complexity on the effectiveness of AIS is essential for firms seeking system optimization.

In 1991, Barney proposed the resource-based view (RBV) and suggested that unique resources and capabilities serve as drivers of competitive advantage [43,44]. AISs are multifunctional, and complexity is considered a valuable, rare, and challenging-to-replicate asset [45]. Complexity enhances the efficiency, flexibility, and reliability of accounting tasks, subsequently improving overall effectiveness [46]. According to institutional theory, organizations tend to conform with societal norms and rules. Complex AISs adapt to continuously evolving accounting standards and regulations, and they help maintain the legitimacy of the system while improving its effectiveness [33,47]. Complex AIS also fosters transparency and accountability, as it involves alignment with stakeholder expectations, thus enhancing the firm's reputation [3]. Meanwhile, according to agency theory, principal–agent dilemmas are alleviated by contract design. Complex AIS provides comprehensive financial information, allowing performance measurement and control to be improved while reducing agency cost [48,49]. It also bolsters overall organizational performance by improving the risk assessment and decision making of firms [38,50].

Several scholars have examined the influence of system complexity on AIS effectiveness. Complex AIS negatively influences the performance of small- and medium-sized enterprises [2,9]. Furthermore, a highly complex AIS may lead to errors and poor accounting [13,51]. Therefore, the complexity of AIS can adversely affect organizational performance. However, other studies have suggested a much more nuanced relationship between AIS complexity and effectiveness. The complexity of AIS is beneficial in certain situations, such as when it allows for much greater system customization to meet the organization's needs [11,12]. Moderate levels of AIS complexity also improve decision making and organizational performance [11,52].

Despite mixed findings, several researchers suggest that organizations must optimize the complexity of their AISs. Gil et al. [52] found that firms tend to balance simplicity and complexity as a way of ensuring that the AIS can still meet their users’ needs. Organizations should also reduce unnecessary complexity in AIS to improve the effectiveness of these systems [3,4,38].

In summary, the relationship between AIS complexity and effectiveness depends on different factors, such as organization size, industry type, and customization needs. High complexity can negatively affect effectiveness, but moderate complexity is regarded as beneficial. Firms must optimize the complexity of their AISs to ensure the effectiveness of these systems. The following hypothesis is proposed.

H1 AIS complexity negatively affects AIS effectiveness.

### 2.2 Involvement of managers in AIS

Businesses continuously hurdle situations of growing complexity; meanwhile, the effectiveness of AIS relies heavily on the degree of managerial involvement. Grounded in the RBV, which postulates that unique resources, such as managerial expertise, can forge lasting competitive advantage, firms can benefit from managers who are knowledgeable about AIS [44,53]. Managers with advanced skills in navigating AISs can align such systems with their respective firms' strategic objectives, thereby increasing their utility [6,54]. Institutional theory supports this view, highlighting organizations' importance in aligning firm objectives with prevailing norms, rules, and standards to secure legitimacy and survival [47]. Managers entrenched in AIS operations can ensure that the system adheres to external standards, further augmenting perceived effectiveness [5,55]. Meanwhile, agency theory underscores the responsibility of managers to act in the company's best interests [56]. Through the active engagement of managers in AIS operations, they can promote transparency and efficiency while restraining potential information asymmetry, thereby strengthening the effectiveness of AISs [38,57].

The involvement of managers in AIS operations is essential for ensuring firms’ effectiveness. When managers take part in designing, implementing, and maintaining an AIS, they enhance their understanding of the data generated by the system and can use these data for decision making [6,18,34,40]. However, the relationship between managerial involvement and AIS effectiveness must be clarified.

The involvement of managers in AIS operations positively affects AIS effectiveness. Al-Dalabih [40] found that managers involved in designing and implementing AIS improve decision making and organizational performance. Moreover, managers' participation in AIS operations increases user satisfaction and enhances the understanding of the system's capabilities [58]. Although Nouir and Samim [9] found that managerial involvement in AIS operations positively affects effectiveness, excessive involvement can decrease effectiveness, mainly because of micromanagement and excessive control. The level of managerial involvement in AIS operations also presents a U-shaped relationship with system effectiveness. Low and high levels of involvement are associated with low levels of effectiveness, whereas moderate levels of involvement are associated with high levels of effectiveness [25,59].

In summary, the involvement of managers in AIS operations is critical to AIS effectiveness. The excessive involvement of managers can lead to decreased effectiveness, but moderated involvement improves decision making, performance, and user satisfaction in organizations. The following hypothesis is proposed.

H2 Managers' involvement in AIS positively affects AIS effectiveness.

### 2.3 Managers’ AIS knowledge

In the dynamic business world, the success of AIS relies on managers' understanding of these systems. From an RBV perspective, managerial insights into AIS are regarded as unique organizational assets that can foster the competitive advantage of firms. Managers with extensive knowledge of AIS can steer strategic decisions, subsequently improving organizational performance and AIS effectiveness [44,46,49]. This viewpoint is reinforced by institutional theory, which requires organizations to conform with prevalent norms and processes to ensure legitimacy [33]. Managers who are knowledgeable about AIS can ensure its harmony with external regulations and accepted norms, further amplifying its effectiveness [22,55,60]. Meanwhile, agency theory posits that managers need to act in the company's best interest, implying that a deep understanding of AIS allows managers to ensure information transparency, reduce agency costs, and improve AIS effectiveness [50,57].

Managers must understand AIS to effectively use these systems. Knowledge of AIS of managers affects the effectiveness of the AIS. Managers with a good understanding of AIS can use the data generated by the system to make informed decisions and identify opportunities for improvement [22,23,61].

Knowledge of AIS among managers is positively associated with the effectiveness of AIS. Managers' knowledge of AIS is positively related to the successful implementation of enterprise resource planning systems [62]. The perceived usefulness and ease of use of AISs are positively associated with the knowledge of AIS of users [63]. However, the relationship between managers’ AIS knowledge and system effectiveness may be much more complex. While AIS knowledge is positively associated with system effectiveness, this relationship may be moderated by the level of IT infrastructure and user satisfaction [58,64]. Furthermore, the relationship between AIS knowledge and effectiveness is mediated by the quality of the data generated by the system [65,66].

Managers’ knowledge of AIS is important for ensuring the effective functioning of this system. While other factors, including IT infrastructure, user satisfaction, and data quality, may influence this relationship, a positive correlation exists between AIS knowledge and system effectiveness [4,61]. Thus, we hypothesize the following.

H3 The AIS knowledge of managers positively affects AIS effectiveness.

### 2.4 Managers’ knowledge of accounting

Managers' knowledge of accounting principles and practices is vital for the effective use of AIS. With a good understanding of accounting, managers can use AIS data to make informed decisions and improve organizational performance. The RBV emphasizes the value of unique resources, such as managerial accounting knowledge, in securing a competitive edge. Thus, adept managers can optimize the use of AIS and align it with strategic needs. Institutional theory emphasizes the criticality of adhering to societal norms and regulations to ensure organizational legitimacy. Therefore, managers with accounting knowledge can enhance AIS legitimacy and efficiency by ensuring compliance with pertinent standards. Meanwhile, agency theory, which focuses on the principal–agent relationship, mandates that managers act in the owners' best interests. Managers’ accounting expertise reduces the possibility of information asymmetry, thereby enhancing AIS transparency, cutting agency cost, and increasing AIS effectiveness.

The knowledge of accounting of managers is related to AIS effectiveness. Managers' knowledge of accounting is positively related to the effective use of AIS [4] and is positively associated with the accuracy and timeliness of financial reporting [2,67,68]. However, the relationship between accounting knowledge and AIS effectiveness may be more complex. The level of the relationship's internal control moderates the relationship's financial reporting quality [23,25,61]. The relationship between accounting knowledge and AIS effectiveness is also mediated by the level of management support when using AIS [3,34].

In summary, the knowledge of accounting of managers is essential for ensuring the effectiveness of AIS. The relationship may be moderated or mediated by factors such as internal controls and management support. However, accounting knowledge is positively related to AIS effectiveness. Therefore, the following hypothesis is proposed.

H4 The accounting knowledge of managers positively affects AIS effectiveness.

### 2.5 Moderating role of external consultants

In business operations, external consultants contribute to the optimization of AIS. The RBV emphasizes the importance of unique resources, such as the specialized knowledge and skills of external consultants, in securing competitive advantage. This aspect highlights that expertise can enhance AIS effectiveness by optimizing the system design, implementation, and utilization and aligning the system with the firm's strategic objectives. Institutional theory, which focuses on conformity to societal norms and regulations to ensure organizational legitimacy, underscores consultants' role in bringing industry best practices and regulatory norms to the firm level, enhancing the alignment of the AIS with standards and increasing its effectiveness. Meanwhile, agency theory, which focuses on the relationship between principals (owners) and agents (managers) and potential conflicts of interest, reveals the importance of consultants acting as neutral third parties. They can ensure AIS transparency and reduce information asymmetry, further enhancing AIS effectiveness.

External consultants are an essential factor in AIS effectiveness. They often seek to provide expertise and support in the implementation and maintenance of AIS, and their role in this process affects AIS effectiveness [4,29,31,32].

Seeking external consultants in AIS implementation and maintenance is positively associated with AIS effectiveness [25,52,59]. External consultants provide expertise and support for the successful implementation and functioning of AIS (A [5]). In the context of a moderating relationship between AIS complexity and AIS effectiveness, external consultants can provide guidance and support in reducing this complexity, leading to improved AIS effectiveness [9,11]. In terms of the moderating relationship between the managers’ involvement in AIS operations and AIS effectiveness, external consultants can help managers perform their operational tasks efficiently, which leads to improved AIS effectiveness [3,58,64]. External consultants can also improve the knowledge of AIS and accounting of managers by providing training and guidance in software and hardware systems and the affirmation of accounting principles, subsequently improving AIS effectiveness (A [5]).

In summary, external consultants are important for the successful implementation and functioning of the AIS. The moderating role of external consultants can improve AIS effectiveness in the context of factors such as AIS complexity, managerial involvement in AIS operations, and managers’ knowledge of AIS and accounting. Hence, the following hypothesis is proposed.

H5 External consultants moderate the relationships between AIS effectiveness and complexity, managers' involvement, managers' AIS knowledge, and managers' accounting knowledge.

**Fig. 1:**
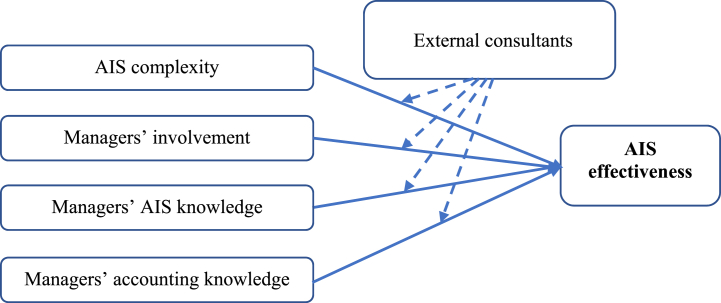
Conceptual research model.

On the basis of the abovementioned theories, the complexity of AIS, the involvement and knowledge of managers, and the moderating role of external consultants jointly shape system effectiveness. To address these issues, we present the research model in Fig. 1.

## 3 Research methodology

A quantitative research design with a cross-sectional survey approach was utilized to investigate the moderating influence of external consultants on the relationships between AIS complexity, managerial involvement in AIS operations, managers’ knowledge of AIS and accounting, and AIS effectiveness. According to the literature [69], a cross-sectional survey is a reliable method for collecting data when variables represent a particular time. Thus, this method was applied in this study.

The Likert scale is a widely accepted and reliable method for measuring constructs in survey research [70,71]. Here, a seven-point Likert scale was used to measure AIS complexity, managerial involvement, managers' knowledge of AIS and accounting, external consultants, and AIS effectiveness. A questionnaire sourced from the literature [70,71] was adopted and then developed to integrate relevant constructs [4,5]. AIS effectiveness was measured using six items, while AIS complexity was measured using five items. Managerial involvement was measured using five items, managers' AIS knowledge was measured using four items, managers’ accounting knowledge was measured using three items, and external consultants were measured using four items.

The research sample included accounting managers working in firms in Ben Tre Province, Vietnam. According to the SEM requirements, the minimum sample size must be at least 50 (preferably 100). The ratio of observed and corresponding measurement variables was 5:1; that is, a measurement variable needs at least five observation variables (preferably 10:1 or more) [72]. For partial least squares–structural equation modeling (PLS-SEM) analysis, a sample size of at least 100 is acceptable, as long as the data are multivariate normal and the model is not too complex [73]. Thus, the minimum sample size is determined by the formula n = 5 m, where m is the number of questions in the survey or the number of measurements. In this study, the 27-item survey represented six constructs. The minimum sample size should be 135. Furthermore, the authors added 30% questionnaires to obtain the sample size. Moreover, 173 questionnaires were sent to respondents face-to-face or via email.

A convenient technique for sampling was used to select 173 participants, with two individuals in different positions representing one company, for the study. In addition, there are 123 companies in the agricultural industry, operating in various fields such as trading and exploiting aquatic products, transportation, trade in services, commerce, manufacturing, and agriculture. These companies are classified according to the enterprise database of Ben Tre province. In accordance with ethical research practices, informed consent was obtained from all participants prior to their participation in the study. The collected data were analyzed using SPSS 26 and Smart-PLS 4.0. Descriptive statistics and path modeling were conducted to assess the relationships among the constructs. Confirmatory factor analysis was applied to assess the reliability and validity of the measurement model by examining the factor loadings, convergent validity, and discriminant validity of the latent variables. Furthermore, moderation analysis was used to test the interaction effects between the determinants and external consultants on AIS effectiveness. The bootstrapping technique was used to test the significance of the path coefficients.

## 4 Results of research

A total of 170 questionnaires were received. Survey respondents were directors, chief accountants, and general accountants. After collecting and examining the questionnaire, three copies were eliminated due to a few answers in the survey being vacant. The authors collected 167 valid responses to analyze the data through SPSS 26 and Smart-PLS 4.0 [74] software. Table 1 provides the demographic profile of the respondents.

**Table 1:** Respondents’ profile.

Demographic characteristic		%
**Gender**	Male	38.3
	Female	61.7
**Position**	Director	16.8
	Vice Director	19.2
	Chief Accountant	19.2
	General Accountant	44.9
**Age**	Under 30 years	20.4
	30–40 years	62.9
	41–50 years	12.0
	Above 50 years	4.7
**Academic**	Undergraduate	84.4
	Graduate	15.6
**Experience**	Under 5 years	11.4
	5–10 years	37.7
	11–15 years	31.7
	Above 15 years	19.2

Note: The number of respondents was 167.

The demographic characteristics of the respondents, comprising a total of 167 participants, provided valuable insights into the composition of the study sample. In terms of gender distribution, the majority of respondents were female, constituting 61.7%, compare with the male, accounted for 38.3%. Regarding the positions, the distribution was diverse, with the highest representation being General Accountants at 44.9%, followed by Directors, Vice Directors, and Chief Accountants at 16.8%, 19.2%, and 19.2%, respectively. The age range of the participants reflected a varied workforce, with the majority falling within the 30–40 years range (62.9%), followed by those under 30 years (20.4%), 41–50 years (12.0%), and above 50 years (4.7%). Academically, a significant proportion of respondents hold undergraduate degrees (84.4%), while 15.6% reported having graduate-level education. With professional experience, the survey captured a diverse range, with respondents having under 5 years (11.4%), 5–10 years (37.7%), 11–15 years (31.7%), and above 15 years (19.2%) of experience. This comprehensive demographic profile contributed to a better understanding of the study sample and created the validity and credibility of following data analysis results.

Afterward, we used SPSS version 26.0 to process the collected data's descriptive statistics and reliability analysis and assess the sample's demographic profile. The internal consistency of the constructs of the research model was tested. Partial least squares (PLS) analysis was implemented on Smart-PLS 4.0. In accordance with the recommended two-stage analytical procedures for SEM, the validity and reliability of the measures of the measurement model were tested, and then the structural model was examined [75,76]. A bootstrapping method (5000 resamples) was used to evaluate the significance of the path coefficients and the loadings. PLS-SEM is suitable for exploratory research and when small sample sizes and complex causal models are used, whereas CB-SEM is appropriate for confirmatory research and when large sample sizes and models with a high degree of measurement error are utilized [75]. Therefore, PLS-based SEM was used in this study.

Table 2 demonstrates the descriptive statistics of the mean, standard deviation, Skewness, and Kurtosis of all main variables, which are related to system quality (AISC), information quality (MAIN, MAAK, and MASK), external consultants (ECON), and Accounting Information System Effectiveness (AISE). These descriptive statistics reveal an overview of the central tendency, variability, and distributional characteristics of the data for variables, providing a foundation for the following analysis.

**Table 2:** Descriptives of the main variables of the study.

Variables	Mean	Std. Dev.	Skewness	Kurtosis
External consultants (ECON)	4.864	1.587	−0.730	−0.398
AIS effectiveness (AISE)	5.082	1.379	−1.095	0.528
Managers' AIS knowledge (MASK)	4.994	1.395	−0.688	−0.250
Managers' accounting knowledge (MAAK)	4.880	1.622	−0.672	−0.627
Managers' involvement (MAIN)	5.056	1.427	−0.732	−0.212
AIS complexity (AISC)	4.947	1.469	−746	−0.458

Table 3 shows that all the constructs had high loadings (above 0.750) on their indicators, indicating strong construct validity. An AVE value higher than 0.70 suggests that the construct captures at least 70% of the variance in its indicators, meeting the threshold for convergent validity. CR (rho_a) values higher than 0.8 and CR (rho_c) values higher than 0.9 were above the recommended 0.70, indicating high internal consistency reliability. The Cronbach's alpha value of all constructs was higher than 0.80, which also suggests high internal consistency reliability. These results indicate that all the constructs had high validity and reliability and can be considered robust for further analysis [77].

**Table 3:** Validity and reliability for constructs.

Constructs	Items	Loadings	AVE	CR
AIS effectiveness (AISE)	AIS operates stably, meeting the needs of output information in a timely manner	0.887	0.762	0.950
	AIS ensures the quality of output information that is easy to understand, complete, and useful	0.873		
	AIS ensures the frequency of accessing/providing output information.	0.850		
	AIS helps employees identify and solve problems faster	0.860		
	AIS optimize benefit and objective	0.854		
	AIS brings high user satisfaction	0.911		
AIS complexity (AISC)	The process of selling and collecting money is complicated	0.881	0.789	0.949
	The purchasing and payment process for suppliers is complicated	0.844		
	The human management process is complex	0.888		
	The production/business process is complex	0.890		
	The process of raising, using, and distributing cash flows is complex	0.936		
Managers' involvement (MAIN)	Managers actively participate in identifying the needs of AIS	0.846	0.775	0.945
	Managers involved in the operation of AIS	0.845		
	Managers participate in the choice of hardware and software for AIS	0.826		
	Managers involved in solving problems arising when implementing AIS	0.886		
	Managers involved in planning future AIS development	0.990		
Managers' AIS knowledge (MASK)	Managers are good at office applications (Excel, Word, PowerPoint)	0.758	0.728	0.914
	Managers are good at the software used in AIS	0.893		
	Managers are good at database systems.	0.846		
	Managers are good at information technology application	0.907		
Managers' accounting knowledge (MAAK)	Managers are good at financial accounting	0.903	0.834	0.938
	Managers are good at management accounting.	0.917		
	Managers are good at taxation	0.920		
External consultants (ECON)	Software provider supports the efficient operation of AIS	0.894	0.744	0.921
	Government agencies support the efficient operation of AIS	0.835		
	The accounting-service company supports the efficient operation of AIS	0.848		
	Hardware system consultant supports the efficient operation of AIS	0.872		

The heterotrait–monotrait (HTMT) matrix is a method for assessing discriminant validity that provides a more accurate comparison between the correlations of different constructs. The values in Table 4 represent the HTMT ratio of correlations between the constructs, and the threshold for discriminant validity is less than 0.90 (SEM). The HTMT values for all construct pairs are less than 0.90 (ranging from 0.6437 to 0.8024), indicating discriminant validity between all three constructs. Therefore, all the constructs were distinct and could be reliably measured separately.

**Table 4:** Heterotrait–monotrait (HTMT) matrix.

	ECON	AISE	MASK	MAIN	AISC
ECON					
AISE	0.7841				
MASK	0.6985	0.7473			
MAAK	0.6437	0.7817	0.7569		
MAIN	0.6721	0.7934	0.7055	0.6444	
AISC	0.6739	0.8024	0.7568	0.7175	1.7698

The R^2^ for AIS Effectiveness was 0.787 indicating that 78.7% of the variance in AIS Effectiveness can be explained by the modeled variables. In Table 5, MAIN (ꞵ = 0.244, p < 0.01) and MAAK (ꞵ = 0.284, p < 0.01) were positively related to AIS Effectiveness while AISC (ꞵ = 0.123, p > 0.05) and MASK (ꞵ = −0.020, p > 0.05) were not significant. Thus, H2 and H4 were supported while H1 and H3 were not supported. Next, we assessed the 4 moderating effects and found that the interaction effects of MAIN*ECON (ꞵ = −0169, p < 0.01), MASK*ECON (ꞵ = 0.122, p < 0.05) and MAAK*ECON (ꞵ = −0.204, p < 0.01) were significant while AISC*ECON (ꞵ = 0.047, p > 0.05). Thus, H6, H7 and H8 were supported while H5 was not supported.

**Table 5:** Structural Model Estimates (hypothesis testing).

**Hypothesis**	Relationship	Std Beta	**Std Dev.**	**t-value**	**p-value**	**PCI LL**	**PCI UL**	**f**^**2**^
**H1**	AISC → AISE	0.123	0.081	1.525	0.064	−0.012	0.256	0.02
**H2**	MAIN → AISE	0.244	0.055	4.448	*p* < 00.001	0.152	0.329	0.11
**H3**	MASK → AISE	−0.020	0.067	0.295	0.384	−0.137	0.082	0.00
**H4**	MAAK → AISE	0.284	0.068	4.146	*p* < 00.001	0.179	0.408	0.15
**H5**	AISC*ECON → AISE	0.047	0.083	0.566	0.286	−0.079	0.194	0.00
**H6**	MAIN*ECON → AISE	−0.169	0.067	2.526	0.006	−0.291	−0.070	0.05
**H7**	MASK*ECON → AISE	0.122	0.066	1.840	0.033	0.021	0.239	0.02
**H8**	MAAK*ECON → AISE	−0.204	0.072	2.823	0.002	−0.338	−0.100	0.06

Further to that we ran a PLS-Predict as suggested by Shmueli et al. [78] to test the predictive power of the model. As shown in Table 6 all the errors of the PLS-SEM model were lower than the errors generated by the benchmark LM model thus the conclusion is that our model has a strong predictive power.

**Table 6:** PLS-predict.

		PLS	LM	PLS-LM
MV	Q^2^predict	RMSE	RMSE	RMSE
AISE1	0.598	1.019	1.079	−0.060
AISE2	0.546	1.071	1.166	−0.095
AISE3	0.554	1.125	1.223	−0.098
AISE4	0.530	1.104	1.210	−0.106
AISE5	0.530	1.077	1.164	−0.087
AISE6	0.596	0.949	1.014	−0.065

**Fig. 2:**
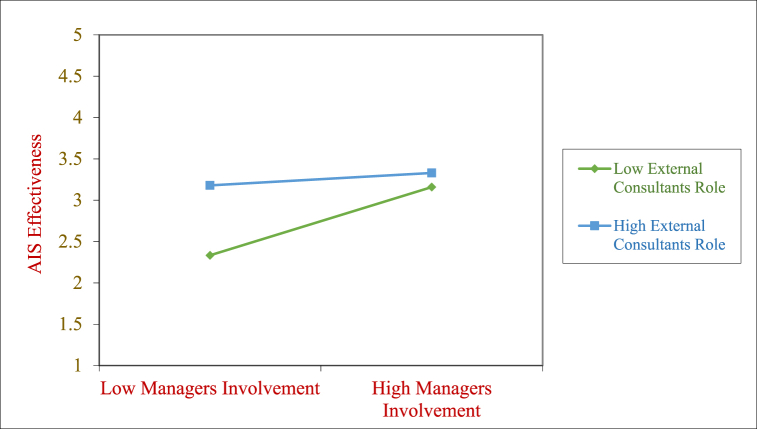
Moderating effect of ECON on the MAIN→AISE relationship.

**Fig. 3:**
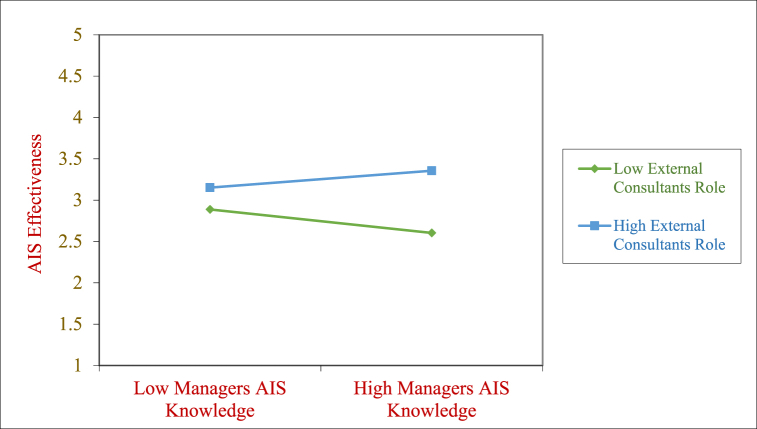
Moderating effect of ECON on the MASK→AISE relationship.

**Fig. 4:**
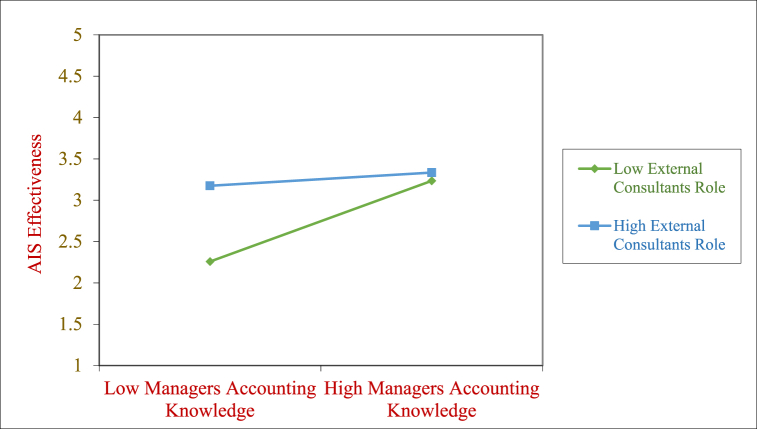
Moderating effect of ECON on the MAAK→AISE relationship.

We next plotted the interaction effects and they are presented in Fig. 2, Fig. 3, Fig. 4. As shown in Fig. 2 the positive relationship between Managers Involvement and AIS Effectiveness is stronger when External Consultant role is lower as compared to when it is higher. This points the resistance of the managers to external consultants getting involved in the implementation when the managers are already highly involved.

In Fig. 3 the relationship between Managers AIS Knowledge and AIS Effectiveness is stronger when External Consultant role is high and it is negative when the External Consultant role is lower. This points the significant role of external consultants’ involvement in the implementation when the managers have low AIS knowledge.

Fig. 4 again we can see that the positive relationship between Managers Accounting Knowledge and AIS Effectiveness is stronger when External Consultant role is lower as compared to when it is higher. This points to the resistance of the managers to external consultants getting involved in the implementation when their Accounting Knowledge is already good.

## 5 Discussion

The involvement of managers in AIS operations and accounting knowledge acquisition significantly and positively affects the AIS effectiveness of firms in Ben Tre Province, Vietnam. The better these factors are managed, the more effective the AIS is. These findings are consistent with those of previous studies that identified the aforementioned factors as essential determinants of AIS effectiveness [4,5,61]. Moreover, as most businesses in Ben Tre Province are SMEs, these factors are crucial to determining AIS effectiveness [2,8]. The active participation of managers of these firms and their knowledge of accounting, as well as the design, construction, and operation of AIS, help to enhance system effectiveness and efficiency [9,40,58]. Limitations in the integration of AIS and accounting knowledge cause managers’ involvement to reduce AIS performance [5,23,25,61].

External consultants moderate the relationships between AIS complexity, managers’ involvement and between their knowledge of AIS and accounting. However, this finding does not manifest in the relationship between AIS complexity and effectiveness. External consultants play a crucial role in optimizing AIS and helping firm managers in Ben Tre Province overcome challenges in utilizing AISs. This result is consistent with the findings of Shahzadi et al. [29], Adil and Ab Hamid, Chen et al. [32], and Chen et al. [25]. In particular, external consultants introduce new perspectives, knowledge, and expertise; these factors complement the existing knowledge of managers and provide guidance, support, and training to managers during AIS operation, thereby enhancing firm effectiveness (A [5]).

The empirical research on Ben Tre Province and the results of hypothesis testing reveal interesting points about AIS effectiveness. Contrary to expectations, AIS complexity (H1) does not significantly impact AIS effectiveness. This deviation could be attributed to a common assumption in numerous management and technology frameworks: that system complexity often acts as a hindrance to effectiveness [43,44]. However, in Ben Tre, AIS is utilized by agricultural firms and businesses offering specific agricultural products. If a system aligns well with the unique needs of businesses in the region, its complexity will not undermine its effectiveness. One possible interpretation was that companies are leveraging technology to bolster performance and competitiveness amid global integration, especially with the onset of ASEAN and ASEAN + agreements. Consequently, they prioritized intricate systems, perceiving them as comprehensive rather than confusing [11,12].

Additionally, the managers' AIS knowledge (H3) did not substantially influence AIS effectiveness. This outcome contrasted with the idea that managerial knowledge about a system bolsters its effectiveness since informed managers can guide system deployment, resolve issues, and align users effectively [4,61]. The discrepancy might arise because AIS effectiveness in Ben Tre depended more on staff proficiency than on managerial knowledge. When end-users were adept with AIS, they rely less on a manager's system expertise. Moreover, the widespread adoption of accounting software and the training of proficient in technology accounting experts ensure that accounting employees are competent users of AIS in firms. This diminished the significance of a manager's AIS knowledge [58,64]. Furthermore, under influence of external consultant, the regression coefficient of MASK*ECON was negative. This implies that when the managers' AIS knowledge increase, it decreases the AIS effectiveness. This result may be intertwined with the factors affecting effectiveness. Potential contributing factors, such as organizational structure, AIS integration with business processes, and complementation of a system to the unique needs of firms in Ben Tre Province, must be considered. A sudden spike in system complexity and lack of organizational adjustment or support mechanisms can eventually reduce effectiveness. This situation highlights the importance of evaluating AIS performance not only on the basis of individual factors but also within the broader organizational context and mutual relationships. The unexpected negative coefficient also emphasizes a nuanced comprehension of the determinants of AIS effectiveness.

The research on firms in Ben Tre Province highlights the disparate roles of external consultants in addressing AIS complexity and effectiveness. The rejection of H5 demands an evaluation of the AIS–consultant dynamics. Although external consultants are pivotal in addressing knowledge and capability gaps, particularly for intricate systems (A [5]), the obtained data from Ben Tre Province suggest a minimal role of consultants in addressing AIS complexity and effectiveness. The contextual adaptation of the AIS seems to simply emphasize technological advancements and the drive toward Industry 4.0 in Vietnam's agricultural sector and Ben Tre Province. The need for a sophisticated system to support agricultural firms in Ben Tre Province can further weaken the relevance of external consultants [2,9]. Moreover, agricultural products, given their seasonal features, have unique attributes that are distinct from those of other industrial products. Given these organizations' particularities, consultants may find it challenging to solve the unique problems relating to AIS [79]. Finally, the ability of company accountants and other end-users to proficiently adapt and utilize AIS might restrict the role of external consultants [28,29,35].

## 6 Conclusion

This study provides insights into the factors affecting the effectiveness of AIS in organizations. The results show that managers' involvement and managers' accounting knowledge positively affect the AIS effectiveness of firms in Ben Tre Province, Vietnam. However, contrasting results were found for AIS complexity and managers’ AIS knowledge. External consultants positively influence AIS effectiveness, but their moderating role in enhancing AIS effectiveness may be hindered by AIS complexity. The contexts in which external consultants are involved in AIS implementation and development must be considered.

This research offers important implications for organizations aiming to improve their AIS effectiveness. For firms in Ben Tre Province, the complexity of their AISs must be carefully considered and then optimized. The first approach is to determine the specific needs and goals of firms through the active involvement of managers in the design, construction, and operation of AIS. The second approach is to enhance the knowledge of accounting and information technology. Firms should also evaluate their external consultants’ potential benefits and limitations in optimizing AIS. While external consultants may offer valuable expertise and resources, their involvement may only sometimes remarkably improve AIS effectiveness.

In the academic realm, this study's contribution to the academic literature is vital because it provides empirical evidence to support the theoretical frameworks and helps further understand the complex dynamics that influence the effectiveness of AIS in organizations. Future research can be conducted to explore additional factors that moderate the relationship between AIS effectiveness and its determinants. For instance, organizational culture, leadership style, and technology infrastructure may be essential in enhancing AIS effectiveness.

While this research sheds light on the determinants of AIS effectiveness in SMEs and the moderating role of external consultants, some limitations should be acknowledged. First, the study was conducted in a specific region which potential limitations associated which the sample's representativeness and needs to be more generalizable to other contexts with various economic and cultural backgrounds. Second, the study focused on a limited number of determinants of AIS effectiveness. It did not explore the potential influence of other factors, such as organizational structure, governance mechanisms, and internal control systems. Finally, causal inferences cannot be drawn because of the nature of cross-sectional research. Future research should address these limitations by adopting a more diverse sample or utilize nationally representative datasets, using alternative research methods, investigating additional determinants of AIS effectiveness, and using longitudinal research designs to establish causality. In addition, the potential endogeneity issues that could influence the causal interpretation of the findings. Thus, future research could consider employing instrumental variables or a fixed-effects approach. Additionally, conducting sensitivity analyses by testing different control variables would provide a more comprehensive understanding of the stability of the results as well as exploring alternative econometric methods, such as panel data analysis or propensity score matching, could strengthen the overall validity of the study.

## Ethics declarations

Review and/or approval by an ethics committee was not needed for this study because we surveyed hand-delivery. It means that all participants know exactly what they need to do and understand their responsibility in answering, and their information will be encrypted and anonymized as an explanation from the authors directly.

All participants provided informed consent to participate in the study. Because the survey is only conducted when the participant agrees and is ready to answer the question.

## Data availability statement

The data that support the findings of this research is available according to the requirement. Restrictions apply to the availability of these data, which were used under license for this study.

For inquiries regarding data access, please contact the corresponding author.

## CRediT authorship contribution statement

**Huu Thien Nguyen:** Writing – review & editing, Formal analysis. **Ramayah T:** Methodology, Data curation, Conceptualization. **Qian Long Kweh:** Writing – review & editing, Supervision, Project administration. **Phuong Thi Kim Tran:** Writing – original draft, Visualization. **Hieu Tran Duong Minh:** Investigation.

## Declaration of competing interest

The authors declare that they have no known competing financial interests or personal relationships that could have appeared to influence the work reported in this paper.
