# Supplementary material for: Determinants of accounting information system effectiveness and moderating role of external consultants: Empirical research in the Ben Tre Province of Vietnam
Source: Heliyon. 2024 Mar 29;10(7):e28847. doi: 10.1016/j.heliyon.2024.e28847 (PMC11004740; doi:10.1016/j.heliyon.2024.e28847)
Supplement: Multimedia component 1 [file mmc1.docx]

**QUESTIONNAIRE**

Dear Mr./Ms.

My name is Tran Duong Minh Hieu, a graduate student majoring in Accounting from Ton Duc Thang University. Today, I am sending you a questionnaire to collect data for my research. I kindly request your attention as your opinions are important to the success of my research.

I undertake and take responsibility for the information collected from you only for the purpose of completing my graduation thesis and I undertake not to use it for any other purpose that affects you or your working unit.

Thank you very much for your help!

**Part 1: Personal information**

Full Name:.............. Email:...........................

Working Unit:............ Telephone:....................

**1. Working position**

☐ Director

☐ Deputy Director

☐ Chief Accountant

☐ Accountant

☐ Other: ...............

**2. Gender**

☐ Male

☐ Female

**3. Age (years)**

☐ Under 30 years

☐ From 31 - 40 years

☐ From 41 - 50 years

☐ Over 50 years

**4. Qualifications**

☐ Undergraduate

☐ Graduate

**5. Work experience**

☐ Less than 5 years

☐ From 6 - 10 years

☐ From 11 - 15 years

☐ Over 15 years

**6. Main fields of operation of your unit**

☐ Production

☐ Commercial

☐ Service

☐ Other: .........

**7. Total capital (VND)**

☐ Less than 3 billion

☐ Over 3 billion to 20 billion

☐ Over 20 billion to 50 billion

☐ Over 50 billion to 100 billion

☐ Over 100 billion

**8. Number of employees**

☐ Under 10 people

☐ From 11 - 50 people

☐ From 51 – 100 people

☐ From 101 – 200 people

☐ Over 200 people

**Part 2: Factors affecting the accounting information systems (AIS) effectiveness in enterprises:**

Instructions for answering: Please mark with an X (or click on the checkbox if using a computer) next to your selected option for levels 1 to 5. Each question has only one choice with the following levels:

(1) Strongly disagree; (2) Disagree; (3) Neutral; (4) Agree; (5) Strongly agree.

| **Content** | | **Response level** | | | | |
| --- | --- | --- | --- | --- | --- | --- |
| **Statement of measurement of factor AIS complexity** | | | | | | |
| *Please indicate your level of agreement with the following statements* | | **1** | **2** | **3** | **4** | **5** |
| 1 | The process of selling and collecting money is complicated | ☐ | ☐ | ☐ | ☐ | ☐ |
| 2 | The purchasing and payment process for suppliers is complicated | ☐ | ☐ | ☐ | ☐ | ☐ |
| 3 | The human management process is complex | ☐ | ☐ | ☐ | ☐ | ☐ |
| 4 | The production/business process is complex | ☐ | ☐ | ☐ | ☐ | ☐ |
| 5 | The process of raising, using, and distributing cash flows is complex | ☐ | ☐ | ☐ | ☐ | ☐ |
| **Statement of measurement of factor Managers’ Involvement** | | | | | | |
| *Please indicate your level of agreement with the following statements* | | **1** | **2** | **3** | **4** | **5** |
| 1 | Managers actively participate in identifying the needs of AIS | ☐ | ☐ | ☐ | ☐ | ☐ |
| 2 | Managers involve in the operation of AIS | ☐ | ☐ | ☐ | ☐ | ☐ |
| 3 | Managers participate in the choice of hardware and software of AIS | ☐ | ☐ | ☐ | ☐ | ☐ |
| 4 | Managers involve in solving problems arising when implementing AIS | ☐ | ☐ | ☐ | ☐ | ☐ |
| 5 | Managers involve in planning future AIS development | ☐ | ☐ | ☐ | ☐ | ☐ |
| **Statement of measurement of factor Managers’ AIS Knowledge** | | | | | | |
| *Please indicate your level of agreement with the following statements* | | **1** | **2** | **3** | **4** | **5** |
| 1 | Managers are good at office applications (Excel, Word, Power point) | ☐ | ☐ | ☐ | ☐ | ☐ |
| 2 | Managers are good at the software used in AIS | ☐ | ☐ | ☐ | ☐ | ☐ |
| 3 | Managers are good at database systems. | ☐ | ☐ | ☐ | ☐ | ☐ |
| 4 | Managers are good at information technology application | ☐ | ☐ | ☐ | ☐ | ☐ |
| **Statement of measurement of factor Managers’ Accounting Knowledge** | | | | | | |
| *Please indicate your level of agreement with the manager's accounting knowledge at your business* | | **1** | **2** | **3** | **4** | **5** |
| 1 | Managers are good at financial accounting | ☐ | ☐ | ☐ | ☐ | ☐ |
| 2 | Managers are good at management accounting. | ☐ | ☐ | ☐ | ☐ | ☐ |
| 3 | Managers are good at taxation | ☐ | ☐ | ☐ | ☐ | ☐ |
| **Statement of measurement of factor External Consultants** | | | | | | |
| *Please indicate your level of agreement with the following statements* | | **1** | **2** | **3** | **4** | **5** |
| 1 | Software provider supports the efficient operation of AIS | ☐ | ☐ | ☐ | ☐ | ☐ |
| 2 | Government agencies support the efficient operation of AIS | ☐ | ☐ | ☐ | ☐ | ☐ |
| 3 | The accounting-service company support the efficient operation of AIS | ☐ | ☐ | ☐ | ☐ | ☐ |
| 4 | Hardware system consultant supports the efficient operation of AIS | ☐ | ☐ | ☐ | ☐ | ☐ |
| **Statement of measurement of dependent factor Accounting Information Systems Effectiveness** | | | | | | |
| *Please indicate your level of agreement with the following statements* | | **1** | **2** | **3** | **4** | **5** |
| 1 | AIS operates stably, meeting the needs of output information in a timely manner | ☐ | ☐ | ☐ | ☐ | ☐ |
| 2 | AIS ensures the quality of output information that is easy to understand, complete and useful | ☐ | ☐ | ☐ | ☐ | ☐ |
| 3 | AIS ensures the frequency of accessing/providing output information. | ☐ | ☐ | ☐ | ☐ | ☐ |
| 4 | AIS helps employees identify and solve problems faster | ☐ | ☐ | ☐ | ☐ | ☐ |
| 5 | AIS optimize benefit and objective | ☐ | ☐ | ☐ | ☐ | ☐ |
| 6 | AIS brings high user satisfaction | ☐ | ☐ | ☐ | ☐ | ☐ |

**Thank you very much for your respond!**

**We wish you a productive day and Respect.**
